# Long-term Outcomes from Proton Therapy for Sinonasal Cancers

**DOI:** 10.14338/IJPT-20-00068.1

**Published:** 2021-06-25

**Authors:** Roi Dagan, Haruka Uezono, Curtis Bryant, Adam L. Holtzman, Christopher G. Morris, William M. Mendenhall

**Affiliations:** From the Department of Radiation Oncology, University of Florida College of Medicine, Jacksonville, FL, USA

**Keywords:** proton therapy, particle therapy, head and neck, outcomes, sinonasal tumors

## Abstract

**Purpose:**

To report long-term disease control, survival, and toxicity after proton therapy for sinonasal cancer.

**Patients and Methods:**

We reviewed 143 cases of adults with nonmetastatic sinonasal cancers treated with primary (18%; n = 26) or adjuvant (82%; n = 117) proton therapy. The most common histologies were squamous cell carcinoma (29%; n = 42), olfactory neuroblastoma (23%; n = 33), and adenoid cystic carcinoma (16%; n = 23). Patients had predominantly advanced-stage disease (T3, 24%, n = 35; T4, 66%, n = 94) and high-grade histology (52%; n = 74). Surgery included endoscopic resection alone (50%) with craniotomy (10%) or open resection (40%), and 31% had gross disease present at radiotherapy. Most (91%) received high-dose (median, 73.6 Gy radiobiological equivalent [GyRBE]; 84% >70 GyRBE) passive-scatter proton therapy using accelerated hyperfractionation (1.2 GyRBE twice daily) and concurrent chemotherapy (70%). Univariate and multivariate models assessed prognostic factors. Grade 3^+^ toxicities were recorded per Common Terminology Criteria, version 4. Median follow-up was 3.4 years (range, 0.1–12.5 years) overall and 4.9 years (range, 0.9–12.5 years) for living patients.

**Results:**

The 5-year outcomes were as follows: local control (LC), 80%; neck control, 96%; local-regional control, 78%; freedom from distant metastases, 71%; and disease-free survival, 62%; cause-specific survival, 64%; and overall survival, 59%. Surgery improved LC, but only with gross total resection (5-year LC 87% versus subtotal resection 62.9%, and biopsy alone 55% (*P* < 0.001). Gross residual disease was the only significant prognostic factor for local-regional control on multivariate analysis. High-grade, T4, and local recurrence were associated with decreased overall survival. Late (G3^+^) toxicity occurred in 22% (32 of 143), including central nervous system necrosis and vision loss in 6% (9 of 143) and 3.5% (5 of 143), respectively.

**Conclusion:**

Proton therapy after gross-total resection provides excellent long-term LC in patients with locally advanced, high-grade sinonasal cancer. Moreover, LC remains strongly associated with long-term survival. With gross disease, about 60% of patients had long-term LC with proton therapy and induction or concurrent chemotherapy.

## Introduction

Sinonasal cancers are some of the rarest and most diverse head and neck malignancies with an annual incidence in the United States of approximately 1 in 180 000 distributed among dozens of histologic subtypes [1–4]. Patients typically present with advanced disease and undergo aggressive multimodality treatment, including surgery, radiotherapy, and chemotherapy, despite the lack of prospective clinical trial data to guide therapy [3, 4]. The proximity of these tumor to (or invasion of) vital structures, including the orbits, skull base, cranial nerves, central nervous system (CNS), and visual pathways, presents a formidable challenge to aggressive local therapy. In most studies, local recurrence is the major mode of treatment failure with rates between 30% and 50%, despite surgery and adjuvant 3-dimensional conformal or intensity-modulated photon radiotherapy (IMRT) [1, 5–7], and results with primary radiotherapy are generally more disappointing [8, 9]. With few patients undergoing successful salvage therapy, local recurrence is the leading cause of death among this patient population.

Charged particle therapy, including proton radiation, has increased in popularity for management of this disease site because of its dosimetric advantages [10, 11] with encouraging early outcomes from single- and multi-institutional cohorts [4, 12–14]. A recent meta-analysis and systematic review suggested disease control and survival benefits with proton therapy compared with IMRT [15]. However, limited data are available to assess long-term outcomes. Therefore, we undertook this study to update our initial experience with proton therapy for sinonasal cancers [4], and here, we report long-term disease control, survival rates, major toxicities, and prognostic factors among nearly double the patients, with double the follow-up, of our previous analysis [4].

## Materials and Methods

Under an institutional review board–approved study, we reviewed the medical records of patients with sinonasal cancers treated with curative-intent primary or postoperative proton radiotherapy at our institution between 2007 and 2018. We excluded patients treated with palliative intent, pediatric patients (younger than 18 years), and those with noncarcinoma histology (mucosal melanoma, bone/soft-tissue sarcoma, and lymphoma), distant metastases, history of prior head and neck cancer or active second malignancy (other than nonmelanoma skin cancers), and any patient with fewer than 6 months of potential follow-up after completion of radiotherapy. In total, 143 patients were eligible for analysis. Staging at the primary site included endoscopy and imaging (computed tomography [CT] and/or magnetic resonance imaging [MRI]). Regional lymph node metastases were staged using neck CT or fluorodeoxyglucose–positron emission tomography/CT (PET/CT), and distant metastases using chest x-ray, chest CT, and/or fluorodeoxyglucose PET/CT.

We previously described our proton therapy technique [4], which is summarized as follows: patients underwent CT simulation in an immobilized supine position on a base-of-skull frame with a 5-point thermoplastic mask, custom neck or occiput support, and shoulder restraints. We used custom oral obturators or stents and dental impressions whenever tolerable for additional immobilization and healthy tissue displacement. We acquired axial images at 1-mm intervals from the vertex to carina with and without intravenous (IV) contrast. We registered preoperative and postoperative imaging (CT and/or MRI) when applicable. We generated a gross tumor volume (GTV) or preoperative GTV based on imaging and operative, endoscopic, or pathologic findings. The standard-risk clinical target volume (CTV1) included the entire operative bed and sinonasal region at risk for subclinical disease and the adjacent skull base. When applicable, we included cranial nerve tracts, orbital and intracranial volumes, and any elective nodal regions in the upper neck in CTV1. The GTV and preoperative GTV to CTV1 expansion was thus anisotropic and varied widely on any given axial slice. We contoured a boost volume, CTV2 (CTV high-risk), as a customized 0- to 5-mm expansion of the GTV or preoperative GTV, which we confined to CTV1. Planning target volume (PTV) margins were 3 mm. The PTV margins were used in aperture design and target coverage evaluation. We contoured the following organs at risk (OARs): globes, optic nerves, chiasm, spinal cord, lenses, lacrimal glands, brainstem, salivary glands, oral cavity, larynx, pharyngeal constrictors, brain, temporal lobes, hippocampi, hypothalamus, pituitary, and brain as healthy tissues at risk.

The proton therapy technique evolved over the study period, marked by a transition from passive-scattered 3-dimensional conformal proton therapy to pencil-beam scanning intensity-modulated proton therapy (IMPT-PBS); however, most patients (127, 89%) received double-scattered proton therapy. Beam arrangements and aperture margins were customized. We used 3 to 4 nonopposed, noncoplanar fields with range modulation to ensure that the spread-out Bragg peak (90% of the mid-spread-out Bragg peak dose) covered the entire radiographic depth of the target. We combined matched fields to improve conformity around complex target volumes with adjacent OARs. We applied distal and proximal margins to the CTV to account for range uncertainty, as previously described [16]. To minimize uncertainties in the treatment planning process, including dose heterogeneity and relative biological effectiveness, we used the minimum allowable field junctions and fields with distal Bragg peaks ending on high-priority OARs, such as the spinal cord, brainstem, or visual pathways [17]. Custom compensators and apertures further maximized dose conformity and reduced the effects of tissue heterogeneity. Compensator smoothing or smearing mitigated the effect of geometric uncertainties on radiographic depth and proton range. Nine patients (6%) received IMRT for technical or logistical reasons (ie, cyclotron downtime or unavailability) and 7 patients (5%) received IMPT-PBS. For IMPT-PBS, the typical field arrangement included of 3 nonopposed, noncoplanar fields with a single anterior-posterior field, and 2 posterior-lateral-oblique fields. The IMPT-PBS plans were planned with multifield optimization, further optimized with robust optimization with a 3-mm margin, with the goal of covering 99% of the CTV and 95% of the PTV with the prescribed dose. For nearly all plans, at least 1 optic nerve and chiasm were limited to a maximum dose of 55 Gy radiobiological equivalent (GyRBE) to 0.1 mL. Brainstem maximum dose to 0.1 mL was similarly limited, although greater doses were accepted when the high-risk target volume abutted the brainstem. The issue of PTV/OAR overlap in the skull base is a major point of tension in planning aggressive radiotherapy for sinonasal and skull base carcinomas. It is an individualized decision, and we heavily weigh the patient's goals of care. We also consider prognosis, functional status, general health, and family or caretaker input. We ultimately favor target coverage and accept up to prescription doses to OARs when the high-risk target overlaps or abuts the OAR in a high-risk situation, such as a positive margins or gross residual disease. In many of these cases, we have judged that an increased risk of serious complication, such as unilateral blindness, will allow for a necessary, curative therapeutic dose, and we find that many patient's prefer up to a 50% risk of a major complication if it means increasing their chance to achieve tumor control or improve survival. We have reduced the PTV 95% dose volume (*D*_95_) to <95% in only 15% (22 of 143) of patients. The OAR constraints did not differ between patients treated once daily or twice daily.

Most patients (n = 130; 91%) received accelerated hyperfractionated proton therapy (1.2 GyRBE per fraction) twice daily with a minimum 8-hour interval. The dose to PTV1 was 45 to 50.4 GyRBE, and the PTV2 was typically planned to receive an aggregate dose of 74.4 GyRBE; however, fractions and/or target coverage could be reduced to meet OAR objectives and standard fractionation (2 GyRBE/once daily) could be used at the discretion of the treating physician. The median aggregate dose to PTV2 was 73.6 GyRBE (range, 54–74.4) over 62 fractions (range, 30–62) and 84% of patients received >70 GyRBE. Patients with negative margins received slightly lower doses (median, 72.4 GyRBE; 53% received >70 GyRBE), and patients with indeterminate or positive margins were treated similar to those with gross disease. Patients with negative margins received slightly lower doses (median, 72.4 GyRBE; 53% received >70 GyRBE), and patients with indeterminate or positive margins were treated similar to those with gross disease.

In total, 121 patients (85%) received regional nodal irradiation, including 105 (73%) for elective coverage of the N0 neck and 16 (11%) as primary or postoperative therapy for a clinically or pathologically positive neck tumor; 22 (15%) were observed with a cN0 or pN0 neck tumor. For most of the study, we treated the neck with our previously described photon technique to 46 to 50 Gy in 23 to 25 Gy daily fractions [4]; however, in the latter part of the study, we included the elective neck target in CTV/PTV1 using IMPT-PBS. In either case, when the neck target included high-risk disease, we boosted the neck volume in CTV/PTV2.

One hundred patients (70%) received chemotherapy; of which, 75% received concurrent low-dose cisplatin (25–30 mg/m^2^) once weekly during radiotherapy with the goal of radiosensitization. The remaining 25% received various forms of multidrug induction or concurrent regimens before either surgery or primary radiotherapy. Toward the end of the study period, nearly all patients with advanced (T3–T4) high-grade neuroendocrine histology (sinonasal undifferentiated carcinoma [SNUC], small cell carcinoma, or Hyams grade 4 olfactory neuroblastoma) received induction chemotherapy with 2 cycles of cisplatin and etoposide or 3 cycles of Taxotere, cisplatin, and fluorouracil before concurrent chemoradiotherapy.

Image guidance consisted of orthogonal kilovoltage x-rays for each fraction with cone-beam CT weekly or daily in more-recent years. During the latter half of the study period, we used midtreatment verification CT (and MRI when indicated) for most patients. Proton therapy plans were adapted whenever target coverage and/or OAR sparing deviated significantly from the initial plan. For primary radiotherapy, boost volumes (CTV2/PTV2) were adapted to account for initial response to therapy (including any induction chemotherapy) based on the findings of the verification simulation.

We prospectively collected disease control data and toxicity endpoints for all patients eligible for on-site follow-up at intervals of ≤6 months for 2 years, then annually thereafter. Follow-up typically included endoscopic evaluation and imaging (MRI or CT) of the primary site with annual imaging surveillance for distant metastases. We performed remote follow-up for all other patients and, whenever possible, we obtained source documents (ie, imaging, pathology, and clinical records) to document disease control and toxicity. We analyzed the following endpoints: local control (LC), neck control, local-regional control (LRC), freedom from distant metastases, disease-free survival (DFS, events coded as any recurrence and censored at the time of death), overall survival (OS), and cause-specific survival (CSS, any death with disease present or related to a treatment complication). A *local recurrences* was any recurrence within or adjacent to the PTV2, including the involved neural tracts or adjacent dura and skull base. A recurrence was deemed marginal when its epicenter was adjacent to or just beyond the high-dose region. Lymph node recurrences in the neck (defining neck control) were designated regional recurrences, and all other recurrences, including leptomeningeal progression or dermal metastases in the head and neck, were considered distant metastases.

We used SAS and JMP software for statistical analysis (SAS Institute, Cary, North Carolina). The Kaplan-Meier product-limit method provided estimates of OS, CSS, LC, NC, LRC and DFS. The log-rank test statistic provided estimates of statistical significance between the strata of selected prognostic factors. Proportional hazards regression was used for multiple regression of selected prognostic factors on each endpoint; a backward selection procedure provided the most parsimonious final models. We assessed serious, late-grade 3^+^ toxicity using the Common Terminology Criteria for Adverse Events, version 4. We reported the single worst toxicity per patient and all grade 3 to 4 visual or CNS toxicities.

## Results

Table 1 shows the baseline characteristics of the 143 study patients. Most were male with advanced (T4, 66%) tumors of the nasal cavity/ethmoid sinus (78%), and the most common histology was squamous cell carcinoma (29%), followed by olfactory neuroblastoma (23%), adenoid cystic carcinoma (16%), and SNUC (11%). A significant proportion of patients had orbital (41%), cranial nerve (28%), and/or intracranial invasion (43%) including 8 patients (6%) with parenchymal brain invasion; 52% had high-grade histologic subtypes. Fifty-five patients (38%) had previously undergone surgery and were being treated for subsequent recurrences.

**Table 1.** Baseline patient and tumor characteristics and surgical approach (N = 143).

**Characteristic**	**Patients or other values, No. (%)**
Age, median (range), y	60 (18–82)
Presentation	
Primary	88 (62)
Recurrence	55 (38)
Primary site	
Nasal cavity/ethmoid	112 (78)
Maxillary	27 (19)
Frontal/sphenoid	4 (3)
Histology	
Squamous cell carcinoma	42 (29)
Minor salivary carcinoma	42 (29)
Adenoid cystic carcinoma	23 (16)
Adenocarcinoma NOS	10 (7)
Mucoepidermoid carcinoma	6 (4)
Other	3 (2)
Neuroendocrine carcinoma	55 (38)
Olfactory Neuroblastoma	33 (23)
SNUC	16 (11)
Small cell carcinoma	3 (2)
Neuroendocrine carcinoma NOS	3 (2)
Other^a^	4 (3)
T stage	
T1	7 (5)
T2	7 (5)
T3	35 (24)
T4	94 (66)
N stage	
N0	127 (89)
N1	6 (4)
N2	10 (7)
Grade	
G1	16 (11)
G2	33 (23)
G3	74 (52)
N/A	20 (14)
Orbital invasion	
Present	58 (41)
Absent	85 (59)
Intracranial invasion	
Present^b^	62 (43)
Absent	81 (57)
Cranial nerve invasion	
Present	40 (28)
Absent	103 (72)
Surgery	
Curative resection	117 (82)
Biopsy only	26 (18)
Surgical approach, n = 117	
Endoscopic	58 (50)
Endoscopic-transcranial	47 (40)
Open	12 (10)
Extent of resection, n = 117	
Gross total resection	103 (72)
Subtotal resection	14 (10)
Margin, n = 117	
Positive/close	59 (42)
Indeterminate (piecemeal resection)	43 (30)
Negative	15 (10)
Neck dissection	
None	124 (87)
Unilateral	15 (10)
Bilateral	4 (3)
Gross disease at primary site at proton therapy^c^	
Yes	45 (31)
No	98 (69)

**Abbreviations:** NOS, not otherwise specified; SNUC, sinonasal undifferentiated carcinoma.

a Anaplastic carcinoma, sarcomatoid carcinoma, ameloblastic carcinoma, and spindle cell carcinoma.

b Includes 8 patients with parenchymal brain invasion.

c Includes biopsy only, debulking surgery (STR), and GTR with recurrence before radiotherapy; 3 additional patients had gross lymph node metastases noted at time of radiotherapy.

Table 1 also shows the surgical approaches used and the resection outcomes. After combining patients who underwent biopsy alone, subtotal resection, and recurrence at the primary site after gross total resection (GTR), 45 (31%) had gross disease at the primary site at the start of proton therapy.

**Figure 1.**
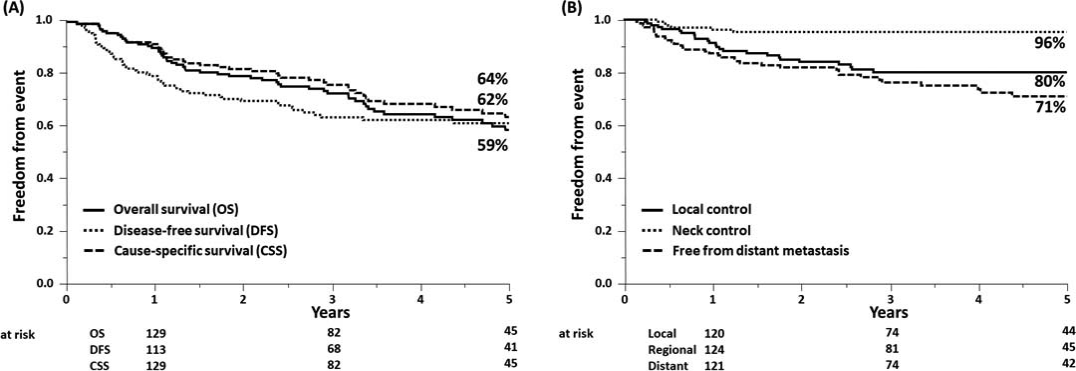
Kaplan-Meier estimates for survival (a) and disease control (b) among the 143 patients included in the analysis.

The median follow-up from the start of radiotherapy was 3.4 years (range, 0.4–7.2 years) for all patients and 4.9 years (range, 0.9–12.5 years) for surviving patients. At the time of analysis, 52 patients (36%) had died, including 37 (26%) from disease progression, 10 (7%) from intercurrent disease, and 5 (3%) directly or potentially because of treatment toxicity. **Figure 1A** shows the Kaplan-Meier curves and estimates for the major survival endpoints. At 3 and 5 years, the OS rates were 73% and 59%, CSS rates were 76% and 64%, and DFS rates were 64% and 62%, respectively.

**Figure 2.**
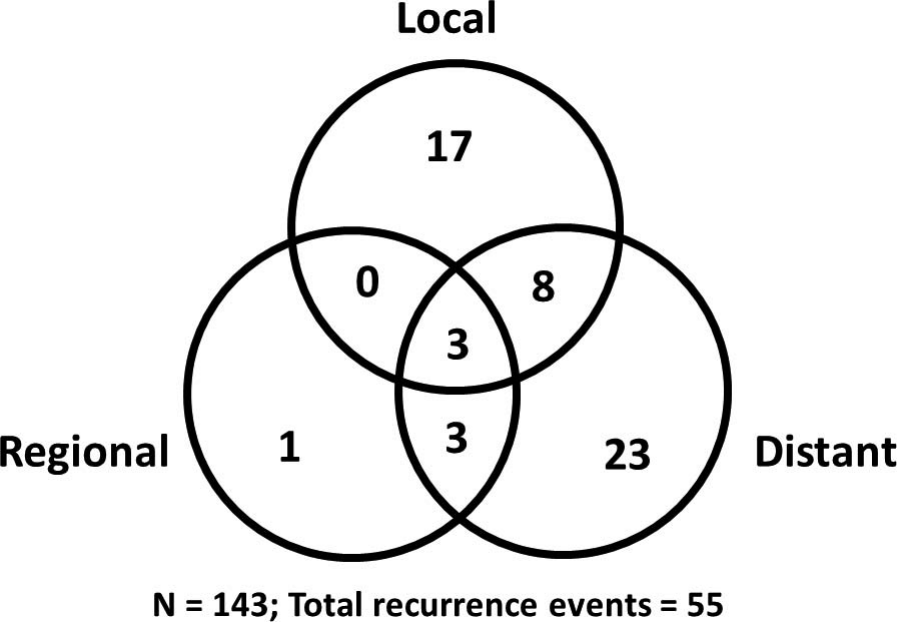
Pattern of progression among the patient population.

**Figure 1B** shows the Kaplan-Meier curves and estimates for the major disease control outcomes. At the time of this analysis, 55 patients (38%) had documented disease progression (Figure 2). The most common pattern of progression was distant metastasis, followed by local recurrence; only 1 patient (1%) had an isolated regional recurrence. The median time to progression for all recurrences was 11.5 months (range, 1.6–115.9 months) with 93% of events occurring within 5 years. At 3 and 5 years, the LC rate was 80% and LRC was 78%. The median time to local-regional progression was 12.7 months (range, 2.7–115.9 months), with 93% occurring within 5 years. Marginal recurrences occurred in 9 patients (n = 6%), and the remainder of the local-regional recurrences were in field. The 3- and 5-year estimates for freedom from distant metastases were 76% and 71%, respectively. The median time to distant progression was also 12.7 months, with 95% of events occurring within 5 years. Of the 4 cases (3%) of progression after 5 years, 2 occurred in patients with adenoid cystic carcinoma, 1 with neuroendocrine carcinoma not otherwise specified, and 1 with SNUC. Leptomeningeal progression was the single most-common site of distant metastases, accounting for 35% of cases with distant progression without local-regional recurrence.

**Figure 3.**
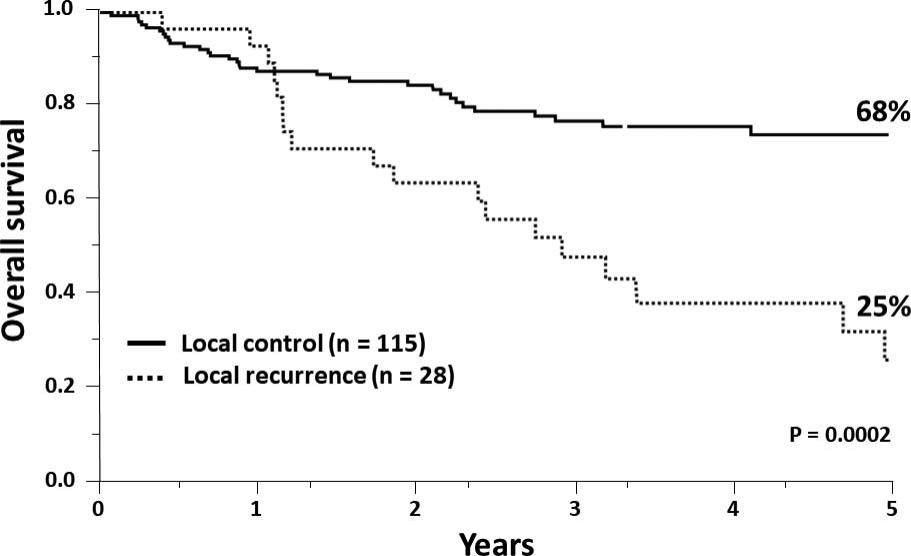
Kaplan-Meier estimates for overall survival with or without local control among the 143 patients included in the analysis.

Table 2 shows the results of our univariate and multivariate analyses of prognostic factors. Nonnasal and ethmoid primary sites, nonsquamous histology, biopsy alone, extent of resection (subtotal resection [STR] versus GTR), and presence of gross disease were associated with lower LRC on univariate analysis with (or trending toward) statistical significance. However, only the presence of gross disease was independently associated with decreased LC on multivariate analysis. An increased risk of distant metastases was associated with histology, T stage, and surgical approach. Along with high-grade histology and T4 disease (versus T1–T3), progression at the primary site was the most significant factor associated with a worse OS on multivariate analysis. From this, we infer that local recurrence remains a major source of mortality despite the competing risks from distant metastases, intercurrent disease, and treatment-related toxicity (Figure 3).

**Table 2.** Results of the univariate (UVA) and multivariate (MVA) analysis.

**Variable**	**Local-regional control**			**Distant metastasis control**			**Disease-free survival**			**Overall survival**		
	**5-y rate, %**	**UVA** ***P*** **value**	**MVA** ***P*** **value**	**5-y rate, %**	**UVA** ***P*** **value**	**MVA** ***P*** **value**	**5-y rate, %**	**UVA** ***P*** **value**	**MVA** ***P*** **value**	**5-y rate, %**	**UVA** ***P*** **value**	**MVA** ***P*** **value**
Site		0.05	0.06		0.45	0.39		0.67	0.60		0.05	0.68
Nasal/ethmoid	83			70			64			65		
Other	62			76			56			39		
Histology		0.32	0.06		**<0.01**	**<0.01**		**0.03**	**0.03**		<0.01	0.08
Squamous cell carcinoma	68			**80**			**65**			52		
Adenoid cystic carcinoma	82			**85**			**77**			89		
Other minor salivary cancer	81			**39**			**38**			29		
Olfactory neuroblastoma	87			**78**			**70**			75		
Other neuroendocrine cancer	77			**62**			**53**			45		
Grade		0.67	0.92		<0.01	0.22		0.02	0.22		**<0.01**	**<0.01**
1	87			90			87			**90**		
2	82			86			76			**73**		
3	72			58			48			**41**		
T stage		0.09	0.17		**0.17**	**0.03**		**0.02**	**<0.01**		**0.03**	**0.02**
T1–%3	84			**75**			**74**			**65**		
T4	75			**69**			**56**			**56**		
N Stage		0.62	0.37		0.35	0.49		0.32	0.53		0.09	0.58
N0	85			68			56			48		
N1–N2	78			72			63			61		
Orbital invasion		0.65	—		0.04	—		0.03	—		0.04	—
No	79			77			69			62		
Yes	79			63			52			56		
Intracranial invasion		0.06	—		0.24	—		0.02	—		0.07	—
No	83			74			70			62		
Yes	71			68			52			56		
Presentation		0.36	—		0.09	—		0.11	—		0.15	—
Primary	77			69			60			58		
Recurrent	100			100			100			78		
Surgery		0.01	0.35		0.52	0.46		0.04	0.30		0.03	0.55
Biopsy only	55			84			45			49		
Curative resection	82			70			65			62		
Surgery extent		<0.01	—		0.92	—		0.22	—		0.41	—
Gross total resection	85			68			66			61		
Subtotal resection	63			83			63			73		
Margin		0.80	—		0.75	—		0.77	—		0.86	—
Positive/close	82			73			68			62		
Indeterminate	83			69			63			64		
Negative	87			67			68			53		
Surgical approach		0.20	—		<0.01	—		<0.01	—		<0.01	—
Endoscopic	85			82			76			73		
Endo/transcranial	61			44			31			50		
Open	83			61			61			52		
Gross tumor at radiotherapy		**<0.01**	**<0.01**		0.67	0.67		**<0.01**	**<0.01**		0.04	0.34
Absent	**87**			70			**68**			63		
Present	**57**			80			**51**			51		
Radiotherapy dose		0.93	0.86		0.99	0.38		0.94	0.57		0.62	0.93
<70 GyRBE	83			73			68			55		
≥70 GyRBE	78			71			62			60		
Chemotherapy		0.09	0.14		0.29	0.80		0.09	0.44		0.04	0.92
No	87			76			72			72		
Yes	74			69			58			53		
Control at primary site		—	—		—	—		**<0.01**	**<0.01**		**<0.01**	**<0.01**
No	—			—			**11**			**25**		
Yes	—			—			**76**			**68**		

Note: Numbers in bold indicate statistical significance.

**Figure 4.**
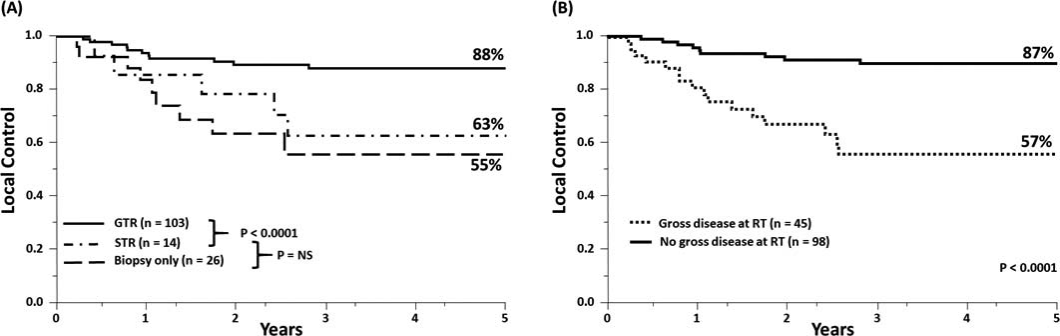
Kaplan-Meier estimates for local control based on extent of surgery (A) and the presence or absence of gross disease (B) at initiation of radiotherapy among the 143 patients included in the analysis. GTR, gross total resection; STR, subtotal resection.

**Figure 4A and 4B** illustrate the effect of surgery and extent of resection on LC. When compared with biopsy alone, surgery appears to improve LC (at 5 years, 85% versus 55%; *P* < 0.01); however, further analysis suggests that the benefit of surgery is limited to GTR because there was no apparent difference between STR and biopsy alone (5-year LC, 63% versus 55%; *P* = not significant [NS]) Ultimately, as noted above, the presence or absence of gross disease at the primary site at the onset of radiotherapy was the most significant prognostic factor for LC.

As mentioned, our practice has trended away from aggressive resection for patients with advanced high-grade neuroendocrine histologic subtypes. Among the 32 patients (22%) treated for SNUC, small cell carcinoma, or Hyams grades 3 or 4 olfactory neuroblastoma, 10 received primary radiotherapy (after biopsy alone or subtotal resection) with and without induction or concurrent chemotherapy. At the time of analysis, the crude LC rate was 80% and 40% were alive without evidence of disease. Of the remaining 22 patients in this category who underwent GTR, the LC rate was 68% and 54% remain alive without evidence of disease. When we exclude olfactory neuroblastoma from this analysis, 13% of patients treated with primary radiotherapy developed a local recurrence compared with 15% who underwent GTR. Although we could not perform a more in-depth statistical analysis because of limited data, these findings suggest that a primary nonoperative approach is appropriate for advanced (T4) high-grade neuroendocrine sinonasal cancers.

Serious late-treatment toxicity occurred in 22% of patients, including 5 deaths directly or potentially associated with treatment (Table 3). Treatment-associated deaths included 3 patients with osteoradionecrosis of the skull base resulting in meningitis or encephalitis, 1 patient with CNS necrosis, and 1 patient who died from pulmonary embolism secondary to a deep vein thrombosis. This patient was nonambulatory because of a fibula free-flap graft-site infection, and she died during adjuvant therapy. The most common grade 3 to 5 events included skull base bone or soft-tissue necrosis with or without associated cerebrospinal fluid leak (11%). Five patients (3.5%) developed unilateral vision loss; all of whom had orbital tumor invasion. We did not include reversible vision loss from cataracts or orbital exenteration as events. Grade 3 to 5 CNS toxicity occurred in 6% of patients. We did not include patients with asymptomatic radiographic changes or minor symptoms that resolved with steroids. Two patients developed treatment-associated tumors, both benign meningiomas. Another patient developed an unknown primary adenocarcinoma metastatic to the liver, which we did not consider to be therapy related. Six patients developed reversible serious grade 3 acute toxicities, including 5 temporary feeding tubes and 1 sepsis.

**Table 3.** Grade 3–5 adverse events per the National Cancer Institute Common Terminology Criteria for Adverse Events, version 4.0. Data are presented as No. (%); N = 143.

**Event/grade**	**All events**	**Ocular/vision loss**	**CNS necrosis**	**Soft-tissue necrosis, ORN, CSF Leak**	**Second tumor**
G3	23 (18)	3 (1)	8 (6)	12 (8)	0 (0)
G4	4 (2)	2 (1)	0 (0)	2 (1)	2 (1)
G5	5 (3.5)	0 (0)	1 (<1)	3 (2)	0 (0)
Total	32 (22)	5 (3.5)	9 (6)	16 (11)	2 (1)

**Abbreviations:** CNS, central nervous system; ORN, osteoradionecrosis; CSF, craniospinal fluid.

## Discussion

This study reports mature outcomes from, to our knowledge, the largest-published single-institution experience with proton radiotherapy for sinonasal cancers. With a median potential follow-up nearing 5 years, we report encouraging LRC (78%) and OS (59%), despite most patients having locally advanced (T3–T4, 90%) and high-grade (52%) disease. We observed more distant progression (∼30%) compared with much of the published literature. However, our findings continue to highlight the importance of aggressive local therapy with a nearly 3-fold benefit in 5-year survival with durable LC, and only 1 in 4 patients alive at 5 years after local recurrence.

Data informing best radiotherapy techniques for management of sinonasal cancers are limited to retrospective research. To date, there is no level 1 evidence, although actively enrolling trials limited by slow accrual are evaluating the role of induction chemotherapy (NCT00707473, NCT03493425). The highest level of evidence addressing radiotherapy techniques derives from a meta-analysis and systematic review of retrospective data, including approximately 1500 patients (∼300 treated with charged particle therapy), which demonstrated improved 5-year LRC, DFS, and OS in a subset of patients treated with proton therapy compared with IMRT [15]. The analysis was limited by the vast heterogeneity among the cohorts, sparse data on actuarial disease control, and a disproportionate ratio of high-risk histologic subtype among the photon cohort (50% versus 27%). Lastly, the reported 20% event rate of CNS toxicity with proton therapy, which was significantly worse than that of the photon cohort, remains a concern. Since the publication of that meta-analysis, additional observational studies have documented favorable outcomes with proton therapy [12–14], which are further supported by our mature results. Given the rarity and diversity of this disease group, high-level evidence is unlikely to emerge, and, thus, using the most conformal radiotherapy technique available to deliver aggressive therapy near critical skull base, CNS, and/or ocular structures is justified. Current National Comprehensive Cancer Center guidelines support the use of proton therapy in this disease site [18], and advances in IMPT-PBS delivery, including incorporation of collimation systems [19], may provide additional dosimetric and clinical benefits. That said, the specific dosimetric benefits or effect of dosimetric uncertainties associated with proton therapy vary from case to case.

We acknowledge that our approach differs from that described in the current literature on radiotherapy for sinonasal cancers in more than radiotherapy technique alone. We remained consistent in our approach in large part so we could better evaluate our outcomes. Our rationale for using dose escalation in the postoperative setting (≥66 Gy), accelerated hyperfractionation (twice-daily therapy), elective regional nodal radiation, and concurrent systemic therapy are described below.

### High-Dose Therapy

Nearly all data on dose-response for postoperative radiotherapy for head and neck cancers are derived from nonsinonasal squamous cell carcinomas treated with photon irradiation; thus, the optimal dose for sinonasal cancers has not been established. Our univariate analysis did not suggest a dose-response >70 GyRBE; however, we had limited data from patients receiving <70 GyRBE, and this analysis is subject to significant confounders and selection bias. Our bias toward high-dose therapy is driven by the observation that most recurrences occur at the primary site despite gross resection and that local recurrence after primary therapy typically leads to death. Moreover, the surgical approach and outcomes in these cancers differ quite drastically from most other head and neck sites, where en bloc resection with radical margins is not limited by the proximity of the critical skull base, orbital, or CNS anatomy. In our experience, patients who undergo minimally invasive, endoscopic/endonasal, piecemeal resection, commonly have positive, close, or indeterminate surgical margins, reflecting broader trends within the surgical literature [3]. In light of the advanced, infiltrative nature of many of these cancers (90% T3–T4 in our experience), at a minimum, the physician directing adjuvant radiotherapy should suspect a relatively high burden of residual microscopic disease in the surgical bed, and gross residual disease is not a rare occurrence. Therefore, we believe the selection of high-dose therapy is justified in most patients.

### Accelerated Hyperfractionation

Although complex, this technique is largely driven by the need for high-dose radiotherapy delivery adjacent to, or overlapping, critical visual-pathway OARs. In nearly all cases of sinonasal cancer, the radiation oncologist is forced to compromise between optimal target coverage and OAR sparing. The available data informing the risk of vision loss from radiation retinopathy and optic neuropathy is suboptimal, but overall suggests that increased dose per fraction (>1.8 Gy/fraction) increases the risk of toxicity, as supported by long-standing principles of radiobiology [20–22]. Although some have suggested an increased relative biological effectiveness with low-dose-per-fraction proton therapy [17], we observed a reassuring 3.5% rate of serious visual pathway toxicity in our cohort despite a significant percentage of patients with orbital invasion and many patients receiving >55 Gy(RBE) to at least one retina and/or optic nerve. This rate of toxicity resembles rates reported with IMRT [6, 15]. Additional benefits from this dose and fractionation strategy include potentially enhanced LC by counteracting the negative effect of tumor cell repopulation during therapy. This strategy may be particularly pertinent with adjuvant proton therapy after incomplete resection or with primary radiotherapy after partial response to induction chemotherapy. Accelerated radiotherapy may help overcome the effect of treatment delays from transferring care to a proton facility or obtaining insurance approval. At least one study has reported that proton therapy is associated with significant treatment delays for patients with head and neck cancer [23].

### Elective Neck Irradiation

Controversial in the management of sinonasal cancers, a more detailed discussion of elective neck irradiation is beyond our scope. With rare exceptions, observing the N0 neck is preferred with low-risk histology (adenoid cystic and other low-grade minor salivary gland carcinoma) and early stage squamous cell carcinoma. However, because the risk for occult regional metastases increases with high-grade histology and advanced disease, often crossing the 10% threshold, elective nodal therapy may be justified. Despite the preponderance of such patients in our study, we observed neck recurrences in <5% of patients, suggesting a benefit to elective neck radiotherapy. Others suggest that high salvage rates for regional recurrences may justify observing the regional nodes [24], even when elective lymph node radiotherapy may be effective at reducing the risk of recurrence [24]. However, our preference is to recommend elective radiotherapy because its associated toxicity is less concerning than toxicity from the primary site treatment.

### Concurrent Systemic Therapy

Our comfort and experience with concurrent low-dose cisplatin with primary or adjuvant photon radiotherapy for head and neck squamous cell carcinoma [25] largely influenced our practice regarding systemic therapy. Chemotherapy was not associated with a benefit in any endpoint in our analysis, a finding almost certainly influenced by confounders and selection bias. Our use of systemic therapy evolved over the study period and varied more than any other treatment factor. Initially, nearly all patients with a good-performance status received concurrent low-dose cisplatin (25–30 mg/m^2^ once weekly), regardless of risk factors; however, more recently, we have favored adjuvant radiotherapy alone in low-risk patients. Conversely, we have intensified systemic therapy in patients treated with primary radiotherapy for unresectable squamous cell carcinoma or high-risk neuroendocrine tumors. In those cases, we recommend a primary approach of induction chemotherapy, restaging, followed by concurrent chemotherapy and proton therapy for patients without progression. Our data in this scenario are limited but suggest that these patients still achieve favorable LC without oncologic resection, which is supported by recent data on SNUC [26].

Given the heterogeneity of these patients and cancers, individualized management seems appropriate. Our findings do not support a one-size-fits-all approach of maximum safe resection followed by adjuvant therapy. Subtotal resection (debulking surgery) did not improve LC over biopsy alone (5-year LC, 63% versus 55%; *P* = NS). Therefore, we only recommend aggressive surgery under reasonable expectation of GTR with acceptable morbidity. Among our cohort, we identified a favorable group of patients at low risk for recurrence (∼90% LRC), including those with no gross disease at the time of radiotherapy, low-grade histologic subtypes, T1 to T3 disease, and negative margins. Especially considering the potential for serious or irreversible toxicity among long-term survivors, these patients are more likely to receive postoperative doses of 60 to 66 GyRBE using standard fractionation (2 GyRBE/fraction once daily), without concurrent systemic therapy. However, in the absence of such favorable risk factors, we remain committed to our approach until compelling data emerge to inform these decisions. Higher-risk patients may benefit from more-intense therapy, including induction chemotherapy, as discussed above, and we should continue to seek innovative systemic therapy for patients at particularly high risk for distant metastases. Currently, the benefits of recent advances, such as immunotherapy with checkpoint inhibitors, remain unclear. Considering the competing risk of distant metastases, patients with high-grade (nonadenoid cystic) minor salivary gland variants may be considered for less-aggressive adjuvant radiotherapy. Additionally, we recommend thorough staging for systemic progression before radiotherapy, including evaluation of the leptomeninges with spine MRI and/or craniospinal fluid sampling in cases of high-grade neuroendocrine histology and intracranial invasion. We previously described this unique pattern of progression [27], and, given overall poor prognosis, such patients may be better served with systemic therapy alone or palliative care, avoiding the unnecessary toxicity of aggressive head and neck radiotherapy. Lastly, the ever-changing landscape of histologic subtypes in sinonasal cancers can inform or confuse our treatment decisions. In recent years, molecular subtyping has identified newly described entities, such as human papillomavirus-related multiphenotypic sinonasal carcinoma [28], which was likely previously classified as adenoid cystic carcinoma. Additionally, recently described entities include INI (SMARCB1)-deficient sinonasal carcinoma [29] and NUT carcinoma [30]. These cancers differ in prognosis, natural history, and response to therapy from their previous classifications, which further challenges our ability to derive treatment decisions from retrospective data. Given that most of our patients underwent surgery at other institutions, it was not feasible to reassess archived specimens for additional molecular subtyping.

Our study has several limitations. As with all single-institution retrospective studies, the data are subject to selection bias, and the lack of randomized comparisons and a heterogeneous patient population limits our ability to draw meaningful conclusions regarding the benefits and effects of each radiotherapy technique, surgical approach, dose and fractionation regimens, and systemic therapy. Moreover, because many patients could not return to our institution for follow-up because they live significant distances from our center, important disease control or toxicity events may be missing from our records. Additionally, the lack of patient-reported toxicities or quality-of-life outcomes limits our understanding of treatment-associated adverse effects. Lastly, we cannot determine how advances in proton therapy (eg, intensity-modulated proton therapy, adaptive proton therapy, or cone-beam CT-based image-guided proton therapy), diagnostic imaging, and surgical techniques may have influenced our outcomes.

## Conclusions

In our cohort of predominantly locally advanced, high-grade sinonasal cancers, proton therapy after gross-total resection provided excellent long-term LC. Even in the presence of gross disease, proton therapy can provide durable LC in nearly 60% of patients. Despite a 30% rate of metastatic progression, LC remains strongly associated with long-term survival. With nearly 1 in 4 patients developing high-grade adverse events, balancing the benefits of LC against significant toxicity remains a challenge. High-grade histologic subtype and T4 disease were associated with increased distant progression, and we recommend a primary nonoperative approach in such patients when a nonmorbid GTR is unlikely.
